# Applicability of spatial early warning signals to complex network dynamics

**DOI:** 10.1098/rsif.2024.0696

**Published:** 2025-05-07

**Authors:** Neil G. MacLaren, Kazuyuki Aihara, Naoki Masuda

**Affiliations:** ^1^ Department of Mathematics, State University of New York at Buffalo, New York, NY 14260-2900, USA; ^2^ International Research Center for Neurointelligence, The University of Tokyo Institutes for Advanced Study, The University of Tokyo, Bunkyo-ku, Japan; ^3^ Institute for Artificial Intelligence and Data Science, State University of New York at Buffalo, New York, NY 14260-2900, USA; ^4^ Center for Computational Social Science, Kobe University, Kobe, 657-8501, Japan

**Keywords:** complex networks, early warning signals, tipping points, dynamics on networks

## Abstract

Early warning signals (EWSs) for complex dynamical systems aim to anticipate tipping points before they occur. While signals computed from time-series data, such as temporal variance, are useful for this task, they are costly to obtain in practice because they need many samples over time to calculate. Spatial EWSs use just a single sample per spatial location and aggregate the samples over space rather than time to try to mitigate this limitation. However, although many complex systems in nature and society form diverse networks, the performance of spatial EWSs is mostly unknown for general networks because the vast majority of studies of spatial EWSs have been on regular lattice networks. Therefore, we have carried out a comprehensive investigation of six major spatial EWSs on various networks. We find that the winning EWS depends on tipping scenarios, although the coefficient of variation and spatial skewness tend to outperform alternative EWSs. We also find that spatial EWSs behave in a drastically different manner between the square lattice and complex networks and tend to be more reliable for the latter than the former. The present results encourage further studies of spatial EWSs on complex networks.

## 1. Introduction

Complex systems display tipping points when there exists some environmental threshold beyond which the system enters a qualitatively different regime [1]. For example, tropical woodland ecosystems may collapse to a relatively barren state as rainfall decreases across a critical threshold [2]. As another example, a novel communicable disease may start to rapidly spread in a population when some environmental conditions are met [3]. Tipping points are in general difficult to anticipate because small changes in driver variables can have markedly different effects on the state of the system [1]. However, a variety of systems display characteristic behaviours in the proximity of a tipping point, and such behaviours have been exploited for developing several early warning signals (EWS) that can anticipate the onset of a tipping point [4–7].

Dynamical systems near a tipping point recover more slowly from a disturbance than those far from a tipping point. This phenomenon, called critical slowing down, leads to increased autocorrelation and variance in time-series data, which are typical EWSs [4]. In fact, to calculate these EWSs, many samples from the same element in the system are required in each environmental condition (e.g. a control parameter value in the case of modelling studies) and over several environmental conditions (i.e. some far from a tipping point and others nearer to it) [6]. For example, when samples independently obey an identical normal distribution, emulating one environmental condition, the sample standard deviation, which is a typical EWS, has a standard deviation proportional to $n^{-1/2}$, where n is the number of samples [8–11]. Therefore, ideally, one wants to secure n=50 samples or more to reliably estimate the sample standard deviation. However, in practice, it is often too costly to collect so many samples per environmental condition [12,13], potentially contributing to the lack of consistent EWSs in empirical systems [14,15]. Furthermore, if n is large, the environment may drift to a different state in the middle of collecting n samples in the field or experiment. If this is the case, the use of the EWS computed from the n samples is compromised because the EWS reflects a range of environmental conditions rather than a single one.

Spatial EWSs seek to mitigate this limitation of ‘temporal’ EWSs by measuring, in each environmental condition, a single sample from many different elements constituting a complex system, rather than obtaining many samples from one element (or multiple elements) in the system [7,13]. We define spatial EWSs as requiring just one sample per element, i.e. n=1. Several proposed spatial EWSs are spatial analogues of temporal EWSs, such as spatial correlation [13], spatial variance and skewness [16], the power spectrum of a state variable of a spatially extended system [17] and recovery length (i.e. as opposed to recovery time) [18]. Other spatial EWSs have no temporal analogue, such as the distribution of patch sizes in patchy environments [19].

EWSs have been probably most vigorously studied for ecological dynamics, many of which take place in physical space. Presumably for this reason, most studies of spatial EWSs have been carried out on spatial regular, grid-like networks modelling two-dimensional ecological landscapes, such as the square lattice with or without periodic boundary conditions [7,19–24] and partial differential equations involving space and time [16,17,20,25–30]. Such a network was also used in a study of EWSs for deforestation transitions [2]. However, many other empirical complex systems for which prediction of tipping points is desired have more complex network structure than regular lattices or two-dimensional continuous planes. Examples include epidemic spreading in human and animal populations [3], progression of diseases in general [5] and symptoms of mental disorders in particular [31,32], and inter-specific population dynamics among animals and plants [33]. Furthermore, even if ecological dynamics occurs in a two-dimensional terrain, habitats may be irregularly distributed and heterogeneous [34,35] such that the underlying network is spatial but heterogeneous. Therefore, empirical studies of spatial EWSs in ecological systems [18,19,27,36–44] may be better justified if spatial EWSs are shown to be valid on heterogeneous networks rather than on regular lattices. However, spatial EWSs have been rarely studied beyond on regular lattices, while notable exceptions exist for complex dynamics models coupling epidemic and opinion dynamics [45,46].

In summary, despite the need, whether or not and which spatial EWSs perform well on heterogeneous networks is largely unknown. In the present study, we comprehensively investigate the performance of six major spatial EWSs on complex networks, compared across dynamics models, environmental parameters, how the tipping point is approached and networks. We also provide mechanistic understanding of why they work or do not work depending on the situation and recommended practices based on our numerical results.

## 2. Methods

### 2.1. Dynamics

We used the following four stochastic dynamics models on networks: a coupled double-well model, a model of mutualistic species interactions, a susceptible–infectious–susceptible (SIS) model and a gene-regulatory model. For the coupled double-well, mutualistic species and gene-regulatory dynamics models, we consider two control parameters: the strength of coupling between nodes, D≥0 and a stress parameter, u, which can exert negative (u<0) or positive (u>0) stress uniformly on all nodes. For the SIS model, we only consider D, which is more conventionally known as the infection rate, as the sole control parameter because the concept of a uniform stress is not realistic for the SIS model. The matrix $A=\left(A_{ij}\right)$ is the adjacency matrix of the network. We assume that the network is connected, undirected and unweighted. Each dynamics is simulated with Gaussian noise $\xi_{i}$ with noise strength σ. The noise applied to different nodes is assumed to be independent of each other.

A coupled double-well model on networks is given by [47]:


(2.1) $$dx_{i}=\left[-(x_{i}-r_{1})(x_{i}-r_{2})(x_{i}-r_{3})+D\sum_{j=1}^{N}A_{ij}x_{j}+u\right]dt+\sigma d\xi_{i},$$

where $x_{i}$ is the dynamical state of the ith node and represents a numeric attribute, such as the species population size or amount of tree cover; $r_{1}<r_{2}<r_{3}$ are parameters which, in the absence of noise and coupling, set the positions of the equilibria and N is the number of nodes. The coupled double-well dynamics has been used for modelling various phenomena, including human social movements [48], interacting biological species [49] and connected climate regions [2]. In the absence of coupling and noise, there are two stable equilibria: a lower equilibrium at $x_{i}=r_{1}$ and an upper equilibrium at $x_{i}=r_{3}$. We set $r_{1}=1$, $r_{2}=3$, $r_{3}=5$, D=0.05 (if the control parameter is u), u=0 (if the control parameter is D) and σ=0.1. Exceptions to parameter values are provided in the electronic supplementary material. We initialize this model in either the lower state with $x_{i}=r_{1}=1$∀i or the upper state with $x_{i}=r_{3}=5$∀i. We consider an ith node to be in the lower state if $x_{i}<r_{2}$ and in the upper state otherwise.

A model of mutualistic species dynamics is given by [50]:


(2.2) $$dx_{i}=\left[B+x_{i}\left(1-\frac{x_{i}}{K}\right)\left(\frac{x_{i}}{C}-1\right)+D\sum_{j=1}^{N}A_{ij}\frac{x_{i}x_{j}}{\tilde{D}+Ex_{i}+Hx_{j}}+u\right]dt+\sigma d\xi_{i},$$

where $x_{i}$ represents the abundance of the ith species, B is a constant incoming migration rate, K is the carrying capacity, C is the Allee constant and $\tilde{D}$, E and H moderate the effect of the interaction term $x_{i}x_{j}$. By following [50], we set B=0.1, K=5, C=1, $\tilde{D}=5$, E=0.9 and H=0.1. We use D=0.05 (if the control parameter is u), u=−5 (if the control parameter is D), and σ=0.001. In the absence of coupling and dynamical noise, this model has a stable lower state with $x_{i}=0$ and a stable upper state with $x_{i}=K$. We initialize this model in the upper state with $x_{i}=6$∀i, which is an arbitrary large value representing an extant population [50]. We start the dynamics from the upper state because a common interest in ecology is loss of resilience in the current species assemblage, which is modelled by collapse from the upper state [4]. We consider that any $x_{i}\leq C$ and any $x_{i}\leq C$ are in the upper and lower state, respectively. To prevent $x_{i}<0$ for any node i and time t, which is not physical for this model, we set $x_{i}=0$ whenever our quadrature algorithm produced $x_{i}<0$ during simulations. We also use the same procedure to prevent $x_{i}<0$ for the following two models.

An SIS model on networks in the stochastic differential equation form is given by [3]:


(2.3) $$dx_{i}=\left[-\mu x_{i}+D\sum_{j=1}^{N}A_{ij}(1-x_{i})x_{j}\right]dt+\sigma d\xi_{i}.$$

The node state $x_{i}$ represents the probability that the ith node is infectious (the ith node is susceptible with probability $1-x_{i}$); D is the infection rate and μ is the recovery rate. We use μ=1 and σ=0.001. In the absence of noise, there is a disease-free equilibrium with $x_{i}=0$∀i, which always exists and is stable when D is below an epidemic threshold value, and an endemic equilibrium in which $x_{i}>0$∀i, which exists and is stable when D is large enough [3]. In the presence of noise, some $x_{i}>0$ are expected at any value of D>0. We simulate this model beginning in either the lower (i.e. almost disease-free) state with $x_{i}=0.001$∀i or the upper (i.e. endemic) state with $x_{i}=0.999$. We consider an ith node to be in the lower state if $x_{i}<5\sigma$ and in the upper state otherwise.

A model of gene-regulatory dynamics on networks is given by [50]:


(2.4) $$dx_{i}=\left(-Bx_{i}^{f}+D\sum_{j=1}^{N}A_{ij}\frac{x_{j}^{h}}{1+x_{j}^{h}}+u\right)dt+\sigma d\xi_{i},$$

where $x_{i}$ represents the expression level of the ith gene, B and f characterize the behaviour of the ith gene in isolation and h controls the interaction of the ith and jth genes. Following [50], we set B=1, f=1 and h=2. We use D=1 (if the control parameter is u), u=0 (if the control parameter is D) and σ=0.001. In the absence of noise, this model has an equilibrium at $x_{i}=0$∀i, which is stable when u or D is small enough and represents the inactive state, and an active state with $x_{i}>0$∀i, which exists when u or D is sufficiently large. We simulate this model from the upper state with $x_{i}=2$∀i because one is often interested in modelling the loss of resilience of the active state [50]. We use the same criteria to define the lower and upper states for the gene-regulatory dynamics as we did for the SIS dynamics.

### 2.2. Networks

We used 30 empirical networks and five synthetic networks built with different generative models. These networks vary in terms of the number of nodes and edges, the heterogeneity of the degree distribution and community structure. Separately, we also analysed a square lattice for comparison purposes. We coerce each network to be undirected and unweighted if it is not and use only the largest connected component. Details of the individual networks are found in the electronic supplementary material, section S1.

### 2.3. Early warning signals

We compare six types of EWS that require only n=1 sample of $x_{i}$ for each node i∈{1,…,N} at any given control parameter value, i.e. Moran’s I, the spatial standard deviation, spatial variance, spatial coefficient of variation (CV), spatial skewness and spatial kurtosis. We compute the EWSs using the equilibrium $x_{i}^{*}$ values defined below. Furthermore, the information required by these EWSs is modest: all but Moran’s I need only the $x_{i}$ values; Moran’s I needs the $x_{i}$ values and the adjacency matrix of the network. We chose these six EWSs because spatial variance is a straightforward variant of the spatial standard deviation and because, apart from the EWSs specific to ecological populations, the other five EWSs are commonly used in the literature.

Moran’s I is defined as follows [51,52]:


(2.5) $$I_{M}=\frac{N}{W}\frac{\sum_{i=1}^{N}\sum_{j=1}^{N}A_{ij}(x_{i}-\bar{x})(x_{j}-\bar{x})}{\sum_{i=1}^{N}(x_{i}-\bar{x}{)}^{2}},$$

where $W\equiv \sum_{i=1}^{N}\sum_{j=1}^{N}A_{ij}$ and x=∑i=1Nxi/N. Moran’s I is a measure of spatial correlation because it quantifies the extent to which neighbouring sampling sites on the same surface or object (i.e. nodes in the case of networks [52]) have similar states [51]. Specifically, $I_{M}$ is the ratio of the normalized cross-products, or covariance, of the node states, ∑i=1N∑j=1NAij(xi−x)(xj−x)/W, to their total variance, ∑i=1N(xi−x)2/N. Moran’s I is similar to Pearson’s correlation coefficient in that it is close to 1 when neighbouring $x_{i}$ have similar values, close to −1 when they are dissimilar and close to 0 when they are not correlated. However, $I_{M}$ can be less than −1 or more than 1 [51].

We refer to the sample standard deviation of the node states as the spatial standard deviation, denoted by s, i.e.:


(2.6) $$s=\sqrt{\frac{1}{N-1}\sum_{i=1}^{N}(x_{i}-\bar{x}{)}^{2}}.$$

Quantity s is the unbiased estimate of the population standard deviation and is often used as an EWS in spatially extended systems [26,39,40]. We also use the sample variance, $m_{2}$, where


(2.7) $$m_{k}=\frac{1}{N}\sum_{i=1}^{N}(x_{i}-\bar{x}{)}^{k}$$

is the kth central moment of x, and the CV. The CV is the normalized variant of s defined by


(2.8) $$\text{CV}=\frac{s}{\bar{x}}.$$

We note that the CV is often used as a spatial EWS [18,36,40,41].

Skewness and kurtosis have been proposed as EWS for time-series data [6], and both have also been used as spatial EWS [16,26,29,39]. Skewness and kurtosis are the scaled third and fourth central moments, respectively, of the probability distribution of a random variable x. Sample skewness is defined as


(2.9) $$g_{1}=\frac{m_{3}}{m_{2}^{3/2}},$$

and quantifies the extent to which extreme values tend to appear to the right (positive) or left (negative) side of the mean and approaches 0 as N→∞ for a symmetric distribution. Skewness may increase (i.e. become more positive) or decrease (i.e. become more negative) as a system approaches a tipping point [7]. Therefore, to ensure that the desired behaviour of $g_{1}$ as a system approaches a tipping point is encoded into a more positive value, we use $g_{1}^{\prime }$ as the EWS, where $g_{1}^{\prime }\equiv g_{1}$ for ascending simulations and $g_{1}^{\prime }\equiv -g_{1}$ for descending simulations (see §2.4 for the simulation protocol and §2.5 for a similar procedure involving Kendall’s τ).

Sample kurtosis is defined as


(2.10) $$g_{2}=\frac{m_{4}}{m_{2}^{2}},$$

and quantifies the magnitude of the extreme values (i.e. how large and how far from the mean) and approaches 3 as N→∞ for a normal distribution. An adjustment $g_{2}^{\prime }=g_{2}-3$ can be used to define excess kurtosis with respect to a standard normal distribution, but we do not use that convention here. Because extreme values should become more common near a tipping point, a larger (i.e. more positive) kurtosis may indicate an approaching tipping point [23].

### 2.4. Simulations

**Figure 1.**
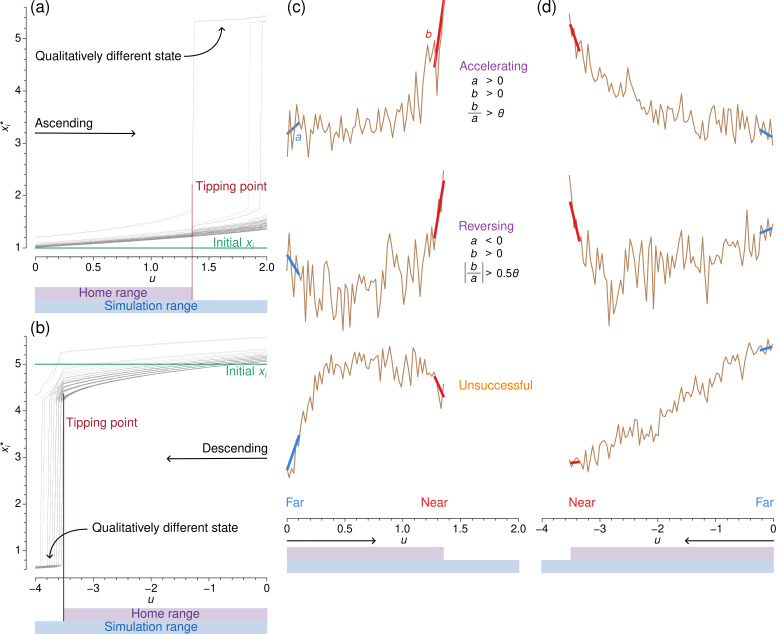
Overview of simulations and classification of EWSs. (a) Ascending sequence of simulations of the coupled double-well dynamics on a Barabási-Albert network with 100 nodes. Each grey line shows the $x_{i}^{*}$ values at a node. When u is small, the $x_{i}^{*}$ are all near their initial value, shown by the green line. As u increases, each $x_{i}^{*}$ tends to increase but stays small until the first tipping point, annotated in red. After the first tipping point, at least some nodes move to a qualitatively different state. The home range of the control parameter is defined to be from the first control parameter value, here u=0, to the control parameter value immediately before the first tipping point. The simulation range contains the home range and encompasses all sampled values of the control parameter. (b) Descending sequence of simulations of the same dynamics on the same network. (c) Three hypothetical EWSs (brown lines) for the ascending simulations shown in (a). We classify an EWS by comparing linear regressions of samples of the EWS taken far from (line a, blue) and near (line b, red) the first tipping point in the home range. We classify the EWS as ‘accelerating’, ‘reversing’ or ‘unsuccessful‘ according to the criteria shown. (d) Three hypothetical EWS for the descending simulations are shown in (b).

We performed simulations for each combination of dynamics model (i.e. double-well, mutualistic species, SIS or gene-regulatory), control parameter (i.e. D or u), direction (ascending or descending; see below) and network (i.e. one of the 35 networks) and measured the performance of the six EWSs. We refer to a combination of a dynamics model, control parameter and direction as the simulation condition. As an example, we consider the coupled double-well dynamics with u as the control parameter initialized in the lower state (corresponding to the ‘ascending’ simulations as explained below), which altogether specifies a simulation condition, on a Barabási-Albert network (figure 1a). We conduct the first simulation with u=0, setting $x_{i,t=0}=1$∀i. We integrate equation (2.1) using the Euler–Maruyama method with Δt=0.01 for 50 time units. We consider $x_{i,t=50}=x_{i}^{*}$ as an equilibrated value under the dynamical noise, collect one sample of $x_{i}^{*}$ from each ith node, and calculate the EWSs. Then, we increase u by a small amount and perform the simulation again using the same initial state. The procedure when D is the control parameter is similar.

We iterate these steps to simulate the dynamics and calculate EWSs at L=100 evenly spaced control parameter values. In the present case, we use 100 values of u in the range [0,2], which we call the simulation range (figure 1a). This range of u results in $x_{i}^{*}\approx 1$∀i when u is smaller than u≈1.35 and at least some $x_{i}^{*}$ in a qualitatively different state, i.e. the upper state (i.e. $x_{i}^{*}>3$), when u is larger.

At large u, the statistics of $x_{i}^{*}$ of the nodes in the upper state are not useful for predicting which nodes will next make the transition from the lower to the upper state [53]. Therefore, we analyse each EWS only for the control parameter values for which all $x_{i}^{*}$ values are near its initial state (i.e. the lower state in the present case). We refer to this restricted parameter range as the home range. As shown in figure 1a, the home range is a subset of the simulation range. Note that the home range is specific to each combination of the simulation condition and network.

We ensured the following two properties in our simulations. First, the simulation range contains at least one tipping point such that the home range is well defined (i.e. the last control parameter value in the home range is just before the first tipping point). Second, the simulation range contains sufficiently many control parameter values near and far from the tipping point such that we can assess the performance of the EWSs as described in the following text. To ensure these two properties, we determined the simulation range by trial and error separately for each simulation condition and network. We show the simulation range for each simulation condition and network in the electronic supplementary material, section S2.

The sequence of simulations shown in figure 1a begins with each $x_{i}$ in the lower state and ends when at least some $x_{i}$ enter the upper state. We call such a sequence of simulations an ascending simulation. By contrast, in figure 1b, we initialize each $x_{i}$ in the upper state, i.e. $x_{i,t=0}=5$∀i, and keep reducing u by a small amount for each of the L simulations. We call this type of sequence of simulations a descending simulation.

### 2.5. Kendall’s τ

As in prior work, we used the Kendall’s rank correlation, denoted by τ, as a performance measure of EWSs [7,13]. We computed τ between the control parameter and the EWS over a range of control parameter values near the tipping event, specifically, the latter half of the home range. We also computed a sign-adjusted Kendall’s $\tau^{\prime }$ value as follows. For dynamics simulated in an ascending sequence, a positive rank correlation indicates that the EWS grows large as the control parameter approaches a bifurcation. In this case, we set $\tau^{\prime }=\tau$. For dynamics simulated in a descending sequence, a good EWS should grow large as the control parameter becomes smaller (more negative). In this case, we set $\tau^{\prime }=-\tau$. A larger $\tau^{\prime }$ value is better. Both τ and $\tau^{\prime }$ range between −1 and 1.

### 2.6. Classification of early warning signals

Kendall’s τ is a dominant performance measure for EWSs but with criticisms [54,55]. Our numerical simulations produced diverse behaviour of the different EWSs as we vary the control parameter, including the case in which the EWS decreases as we approach the tipping point. Given this situation, solely relying on Kendall’s τ would not generate useful comparison between EWSs. Therefore, we developed a classification scheme of EWS as follows.

Consider the example simulation sequence shown in figure 1a, in which we start with the lower state and gradually increase the control parameter, u. Suppose that an EWS responds to the gradual increase in u within the home range of u as shown by the uppermost brown line in figure 1c. This EWS is noisy but remains low when u<1. It then increases progressively rapidly as u approaches the tipping point. This is an ideal case because small values of the EWS reliably indicate that the system is far from transition, whereas large values indicate that the first transition is nearby in terms of u. We quantify the extent to which an EWS follows this pattern using the trend in the EWS value when it is far from the first transition (slope a, blue line in the upper panel of figure 1c) and when it is near (slope b, red line). In the upper panel in figure 1c, both a and b are positive and b is substantially larger than a. We classify an EWS that behaves in this fashion as successful and say that the EWS is accelerating. We will give the precise definition below.

Alternatively, an EWS may first tend to decrease as u increases when u is far from its tipping point, as in the middle panel of figure 1c. However, if the EWS steadily increases at larger u values near the tipping point, this EWS behaviour also reliably indicates that the system is approaching an impending tipping point. Therefore, we also classify this behaviour as successful and say that the EWS is reversing.

Many other trends in EWS behaviour as a function of the control parameter are possible. For example, the EWS value may initially rise rapidly as u increases far from a tipping point. Then, the EWS may level off or even decrease as u further increases, approaching the tipping point, as in the lower panel of figure 1c. Such an EWS gives a false positive while the system is still far from the bifurcation and a false negative when it is close to the bifurcation. We classify such a behaviour, and any other pattern not covered by the accelerating and reversing categories, as unsuccessful.

We classify EWSs in the same manner when we start from an upper state and gradually decrease the control parameter, as we show in figure 1d. The lower panel of figure 1d shows a different case of failure (i.e. neither category) from that shown in the lower panel of figure 1c just for demonstration.

To compute a, we run an ordinary linear least square regression on the first five values of the control parameter from the home range, in which the independent variable is the control parameter and the dependent variable is the EWS. We compute b in the same fashion but using the last five values of the control parameter in the home range. If a and b are positive and b/a>θ, where θ>1, we say that the EWS is accelerating. We set θ=2. If a is negative, b is positive and |b/a|>0.5θ, then we say that the EWS is reversing. We use 0.5θ to capture trends in an EWS that have increased markedly with respect to an initial negative trend without being too strict.

## 3. Results

We ran numerical simulations on four dynamical system models on networks, i.e. coupled double-well, mutualistic species, SIS and gene-regulatory models. We performed sequences of simulations of each model across a range of parameter values, forcing a bifurcation, on 35 empirical and model networks. We then computed the six spatial early warning signals for each simulation sequence. To assess the quality of each EWS, we computed $\tau^{\prime }\in \left[-1,1\right]$, the sign-corrected version of Kendall’s rank correlation. We further classified each EWS as accelerating, reversing or unsuccessful based on the extent to which it showed desirable warning signal behaviour far from and near to the tipping point. We found that the overall trends of the performance results were similar among the three EWSs based on the spatial variance, i.e. s, $m_{2}$ and CV, and that CV outperformed s and $m_{2}$ in most simulations (see the electronic supplementary material, S3, S4 for the results). Therefore, we do not show the results for s or $m_{2}$ in the following text unless we state otherwise.

### 3.1. Examples

**Figure 2.**
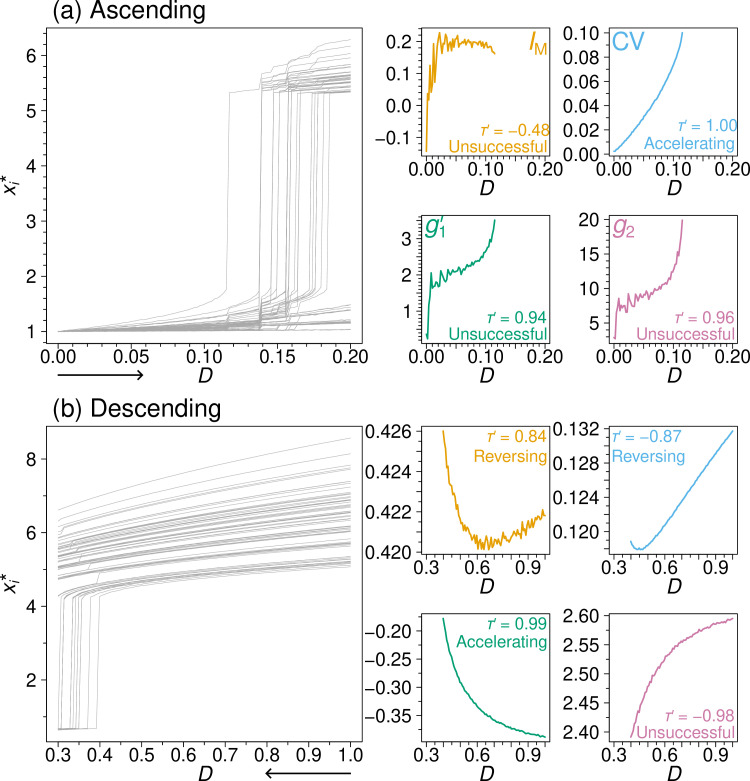
Node states and EWSs as a function of D for the coupled double-well dynamics on the drug interaction network. (a) Ascending simulations. (b) Descending simulations.

As an example, let us consider the coupled double-well dynamics (see equation (2.1)) on the drug interaction network. We use the coupling strength, D, as the control parameter, which we gradually increase starting from zero (i.e. ascending simulations). The grey lines in figure 2a represent the $x_{i}^{*}$ values for all the nodes as a function of D. When D is small, all the nodes are in their lower state (i.e. $x_{i}^{*}<3$). As D gradually increases but is still smaller than the tipping point, each $x_{i}^{*}$ becomes larger but still remains in the lower state. The first transition of any node to the upper state occurs around D≈0.115, and progressively larger values of D result in the transition of more nodes.

The orange line in figure 2a indicates that $I_{M}$ initially increases rapidly, then levels off and even decreases as D approaches the tipping point. This pattern of $I_{M}$ values is not desirable as an EWS and is reflected in the negative sign-adjusted Kendall’s rank correlation value ($\tau^{\prime }=-0.48$); our algorithm classifies $I_{M}$ as ‘unsuccessful.’

By contrast, CV, shown by the blue line, grows in an accelerating fashion as D approaches the tipping point, yielding $\tau^{\prime }=1$ and classification as ‘accelerating’, one of our two successful categories. Both $g_{1}^{\prime }$ (green) and $g_{2}$ (magenta) behave similarly to, while more noisily than, CV, except that $g_{1}^{\prime }$ and $g_{2}$ rapidly increase initially as D increases. Although both $g_{1}^{\prime }$ and $g_{2}$ are desirable EWSs in terms of $\tau^{\prime }$, with $\tau^{\prime }\approx 1$, our classification scheme classifies them as ‘unsuccessful’ owing to their initial rapid increase. However, if the simulation starts from a larger value of D while the last value of D remains the same, we can remove the part of the curve in figure 2a where $g_{1}^{\prime }$ or $g_{2}$ rapidly increases. Then, $g_{1}^{\prime }$ and $g_{2}$ would be classified to be successful. See the electronic supplementary material, S4, for results supporting this phenomenon across different networks. Given such a scenario, we advise to refer to both $\tau^{\prime }$ and our classification scheme in general.

The performance of each EWS depends on the simulation condition (i.e. combination of a dynamics, control parameter and ascending versus descending simulation direction). In figure 2b, we show each $x_{i}^{*}$ when we initialize $x_{i}$ in the upper state and gradually decrease D from D=1 (i.e. descending simulations); the dynamics model and network are the same as that used in figure 2a. In this case, $I_{M}$ initially decreases when D decreases and is still far from the tipping point. Then, $I_{M}$ markedly increases as D further decreases and approaches the tipping point. Overall, the trend of $I_{M}$ values is desirable; we obtain $\tau^{\prime }=0.84$ and classify $I_{M}$ as ‘reversing’, one of the two successful categories.

The other EWSs also perform differently from how they did in figure 2a. In figure 2b, CV performs poorly in terms of $\tau^{\prime }$; its overall trend is largely linear and monotonically decreasing (rather than increasing) towards the tipping point, yielding $\tau^{\prime }=-0.87$. We classify CV as ‘reversing’ because CV increases near the bifurcation. Finally, $g_{1}^{\prime }$ behaves nearly ideally ($\tau^{\prime }=0.99$, classified as ‘accelerating’), whereas $g_{2}$ behaves almost conversely ($\tau^{\prime }=-0.98$, classified as ‘unsuccessful’).

In summary, the performance of the different EWSs can substantially vary depending on the direction of the simulations (i.e. ascending versus descending simulations) although the dynamics model and the network are the same. Therefore, we expect that there are various situations in which one EWS may work better than another and vice versa, which we investigate in the following sections.

### 3.2. Variability in early warning signals performance over simulation conditions

To assess the variation in performance of the different EWSs across conditions, we carried out simulations with each simulation condition on 35 networks.

**Figure 3.**
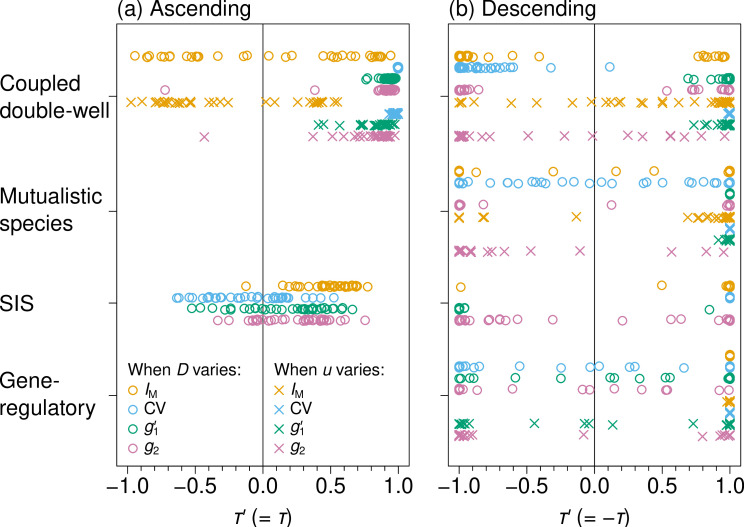
Sign-corrected Kendall's τ values, $\tau^{\prime }$, for four dynamics models, two control parameters (i.e. D, shown by the circles, and u, shown by the crosses) and 35 networks. For each set of simulations, $I_{M}$ is shown in orange, CV in blue, $g_{1}^{\prime }$ in green and $g_{2}$ in magenta. (a) Ascending simulations. (b) Descending simulations.

As an initial analysis, we show the $\tau^{\prime }$ values for each EWS and simulation condition in figure 3. Note that we omitted six simulation conditions as being unrealistic (see §2 ). Therefore, the results for these simulation conditions are missing in figure 3 (i.e. mutualistic species and gene-regulatory in figure 3a and variation in u for the SIS). We find that $I_{M}$ sometimes performs well (i.e. shown by orange markers near $\tau^{\prime }=1$) and sometimes poorly ($\tau^{\prime }$ much smaller than 1, including near −1). There are many intermediate values of $\tau^{\prime }$, particularly for ascending simulations (figure 3a). On the other hand, CV performs remarkably well when the control parameter is u (i.e. stress) and under some other simulation conditions in which the control parameter is D. Skewness $g_{1}^{\prime }$ generally performs well on the coupled double-well and mutualistic species dynamics, and its performance is mixed on the SIS and gene-regulatory dynamics; $g_{2}$ performs best on ascending simulations of the coupled double-well dynamics and has mixed performance otherwise.

**Figure 4.**
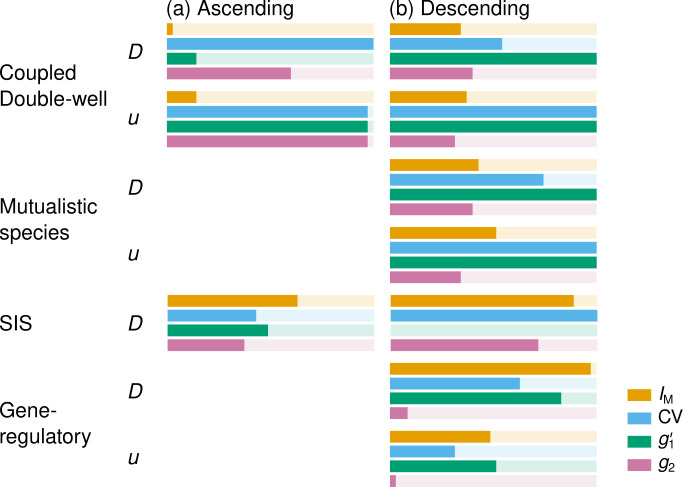
Classification of EWSs for four dynamic models on 35 networks. Solid bars indicate the fraction of networks for which the EWS is successful (either accelerating or reversing category). (a) Ascending simulations. (b) Descending simulations

We applied our classification procedure to further quantify the performance of the different EWSs. The solid colour bars in figure 4 represent the proportion of networks out of the 35 networks with successful EWSs (i.e. classified as either accelerating or reversing) under each simulation condition. The figure supports the observation made in figure 3 that the performance of an EWS depends on the simulation condition. For example, CV is successful for the largest proportion of networks on average among all the EWSs, including $m_{2}$ and s (see the electronic supplementary material, S4 for the results). Instead, $g_{1}^{\prime }$ is the best for the coupled double-well and mutualistic species dynamics in descending simulations regardless of the control parameter. Although figure 4 indicates that $g_{1}^{\prime }$ and $g_{2}$ perform poorly in ascending simulations of the coupled double-well dynamics when the control parameter is D, the performance considerably improves if we start simulations from a larger D value (see the electronic supplementary material, S4 for the results). Moran’s I, on the other hand, is reliable for the SIS and gene-regulatory dynamics in the descending simulations.

Figure 4 suggests that CV and $g_{1}^{\prime }$ perform better than $I_{M}$ and $g_{2}$ overall. To quantitatively investigate the relative performances of the EWSs including s and $m_{2}$, we assign each EWS a score based on its rank: the best EWS among the six EWSs for a particular simulation condition received 5 points, the second best a 4 and so forth and the worst a 0. In the case of a tie, the tied EWSs receive the average score (e.g. if two EWSs are tied for best, both receive 4.5 points). Summing this score across the 10 simulation conditions confirms that CV (32.5 points) and $g_{1}^{\prime }$ (32 points) are almost equally the best, followed by $m_{2}$ (25), $I_{M}$ (23), s (22) and $g_{2}$ (15.5). Overall, CV was categorized as successful in most cases (76.3%), followed by $g_{1}^{\prime }$ (69.4%), $m_{2}$ (54.3%), s (53.7%), then $I_{M}$ (48.0%) and $g_{2}$ (42.3%), as shown in the electronic supplementary material, S4. In terms of $\tau^{\prime }$, it turned out that $g_{1}^{\prime }$ was the best, CV was the second best and the other four EWSs performed notably worse than $g_{1}^{\prime }$ and CV (see the electronic supplementary material, S3 for the results). Therefore, it appears that CV and $g_{1}^{\prime }$ are the most reliable spatial EWSs among the six EWSs.

In §1, we pointed out that most studies of spatial EWSs were carried out on the square lattice or its continuous variants. Therefore, we examined the performances of the spatial EWSs on the square lattice with N=100 nodes and periodic boundary conditions. We find that results for the square lattice are substantially different from those for the 35 networks. Specifically, our algorithm classifies CV as a successful EWS for the square lattice in 8 out of 10 simulation conditions, $I_{M}$ in 5 out of 10 conditions, $g_{1}^{\prime }$ in 2 out of 10 conditions and $g_{2}$ in 1 out of 10 conditions. The failure of $g_{1}^{\prime }$ is in stark contrast with the case of heterogeneous networks. We hypothesized that this last result is because all nodes are structurally equivalent to each other in the square lattice, such that $x_{i}$ values are statistically the same for all i, yielding the lack of strong asymmetry in the distribution of $x_{i}$. We have verified that, under the simulation conditions in which $g_{1}^{\prime }$ is unsuccessful, $g_{1}^{\prime }$ is centred around 0 as the control parameter varies (see the electronic supplementary material, S5). Furthermore, except CV, spatial EWSs on the square lattice are typically noisy, leading to smaller $\tau^{\prime }$ values even when an EWS is classified as successful. For $I_{M}$, the average $\tau^{\prime }$ value is 0.27 when it is successful on the square lattice and 0.86 when it is successful on all other networks. For $g_{1}^{\prime }$, the average $\tau^{\prime }$ value is −0.25 when it is successful on the square lattice and 0.87 when it is successful on all other networks. We show examples of the noisiness of EWSs on the square lattice in the electronic supplementary material, S6. In summary, we conclude that what we know about spatial EWSs on the square lattice does not translate to the case of heterogeneous networks, which further strengthens the need for the present study.

### 3.3. Mechanisms of variable performance of early warning signals

We have shown that different EWSs perform better than others in different simulation conditions. Focusing on heterogeneous networks, we explore mechanisms behind this observation in this section. Because $g_{2}$ was the worst performer in all our comparisons, we do not investigate it further.

#### 3.3.1. Coefficient of variation

**Figure 5.**
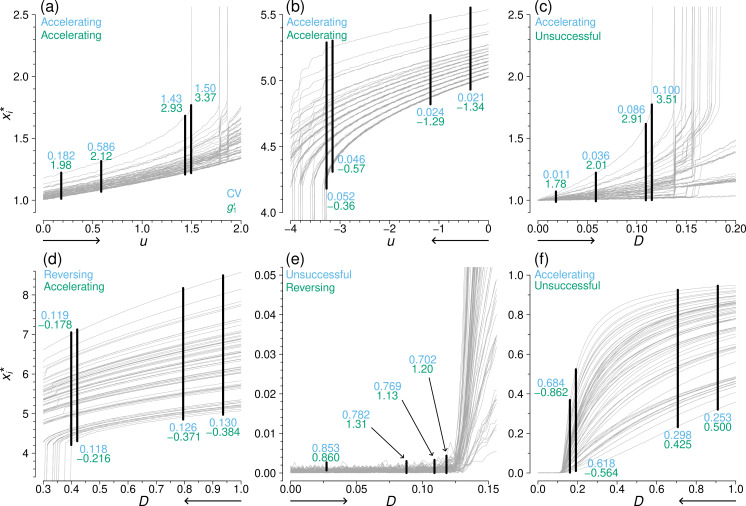
Spatial coefficient of variation (CV) and spatial skewness ( $g_{1}^{\prime }$) as a control parameter varies towards the tipping point. Each grey line shows $x_{i}^{*}$. The spread of the $x_{i}^{*}$ obtained at four control parameter values is shown in black and labelled with the values of CV (blue) and $g_{1}^{\prime }$ (green) at the control parameter values. Classification results are provided in the corresponding coloured text. The direction of the simulation sequence is indicated by the arrow beneath the horizontal axis. We used the drug interaction network. (a) and (b): coupled double-well, with u as the control parameter. (c) and (d): coupled double-well, with D as the control parameter. (e) and (f): SIS, with D as the control parameter.

To examine why CV performs well in some simulation conditions and not in others, let us consider again the coupled double-well dynamics on the drug interaction network used for the demonstration in figure 2. In figure 5a, we gradually increase u and observe $x_{i}^{*}$ and CV (and $g_{1}^{\prime }$). In this case, CV increases in an accelerating fashion as u approaches the tipping point, successfully anticipating the saddle-node bifurcation, which the CV values shown in blue in the figure at four values of u indicate. Qualitatively the same behaviour occurs for all 35 networks. Figure 5a also indicates that the accelerated increase in CV is caused by the behaviour of the $x_{i}^{*}$ values of high-degree nodes (shown by the grey lines with larger $x_{i}^{*}$). These nodes receive more input from their neighbours and thus approach a bifurcation earlier than low-degree nodes do [53]. These $x_{i}^{*}$ values thus separate from those of the smaller degree nodes near the bifurcation, causing CV to increase.

In figure 5b, we show numerical results under the same simulation condition as in figure 5a except that the direction is reversed (i.e. descending simulations). In this case, small-degree nodes separate from the rest as the bifurcation is approached. Nevertheless, their effect on CV is the same as in the case of figure 5a. In other words, the value of CV grows owing to a progressive deviation of the smallest $x_{i}^{*}$ values at small-degree nodes as u decreases towards the saddle-node bifurcation.

When the control parameter is D, the behaviour of CV is not equivalent in both directions. In the ascending simulations, the $x_{i}^{*}$ values of large-degree nodes separate from the remainder (see figure 5c; this is the same simulation condition as in figure 2a), which is similar to when the control parameter is u (see figure 5a). Therefore, CV increases in an accelerated manner, successfully anticipating the bifurcation. However, in descending simulations, the $x_{i}^{*}$ values of the different nodes come closer together as D decreases towards the bifurcation (see figure 5d; also see figure 2b). This result is opposite to the results when the control parameter is u (figure 5b). The reason for this behaviour is in that the input from the other nodes to the ith node, given by $D\sum_{j=1}^{N}A_{ij}x_{j}$, is roughly proportional to the degree of the ith node (i.e. the number of j values for which $A_{ij}=1$). Owing to the multiplicative factor D, as D becomes smaller, the nodes receive smaller and more homogeneous amounts of input from their neighbours. Therefore, the $x_{i}^{*}$ values come closer, reducing CV. Although CV is still categorized as successful in figure 5d, it is because CV starts to increase very near the bifurcation (i.e. roughly between the two leftmost vertical thick lines in the figure) as D decreases. The $x_{i}^{*}$ values behave similarly as D gradually increases or decreases in the SIS dynamics, which show transcritical bifurcations (see figure 5e,f). However, whether or not CV is successful in figure 5e,f does not coincide with the results for the coupled double-well dynamics shown in figure 5c,d, respectively. This discrepancy is probably because CV is a quantity normalized by the mean of $x_{i}^{*}$.

#### 3.3.2. Spatial skewness

The causes of the favourable behaviour of $g_{1}^{\prime }$ as an EWS are related to those of CV as follows.

First, CV and $g_{1}^{\prime }$ are successful in figure 5a,b for the same reason. EWS $g_{1}^{\prime }$ is also successful in figure 5e for the same reason although the increasing trend near the tipping point is weak. Specifically, as the tipping point is approached, $x_{i}^{*}$ of nodes closer to the tipping (i.e. high-degree nodes in figure 5a,e, and low-degree nodes in figure 5b) more rapidly deviate from the remainder, i.e. becoming larger in figure 5a,e, and smaller in figure 5b. Then, the overall variability of $x_{i}^{*}$ grows, captured as an increase in CV as well as $g_{1}^{\prime }$. In this manner, skew increases in ascending simulations at least near the tipping point (i.e. figure 5a,e; increasing $g_{1}^{\prime }$), driven by the large-degree nodes, whereas skew decreases in descending simulations (i.e. figure 5b; this also increases $g_{1}^{\prime }$) as the low-degree nodes separate from the other nodes, making the distribution more symmetric. The overall pattern is the same in figure 5c except that the initial rapid increase in $g_{1}^{\prime }$ causes a misclassification by our algorithm (see figure 2a).

Second, in figure 5d, $g_{1}^{\prime }$ is clearly successful, whereas CV is less so. As noted above, there is a tendency for CV to reverse its decline near the tipping point, while this tendency is weak. By contrast, as the tipping point is approached, the distribution of $x_{i}^{*}$ becomes detectably more symmetric, which successfully increases $g_{1}^{\prime }$. This change is primarily caused by a faster decrease in $x_{i}^{*}$ at the large-degree nodes rather than by that at the small-degree nodes; the latter was the case in figure 5b.

Third, in figure 5f, $g_{1}^{\prime }$ is unsuccessful because $x_{i}^{*}$ is bounded in [0,1] for the SIS dynamics. Owing to this restriction, a faster decrease in $x_{i}^{*}$ at the large-degree nodes as the tipping point is approached, which occurs in the case of the coupled-well dynamics to let $g_{1}^{\prime }$ perform well (see figure 5d), cannot occur in the case of the SIS dynamics. In this case, the behaviour of $g_{1}^{\prime }$ is apparently not related to that of CV.

#### 3.3.3. Moran’s I

**Figure 6.**
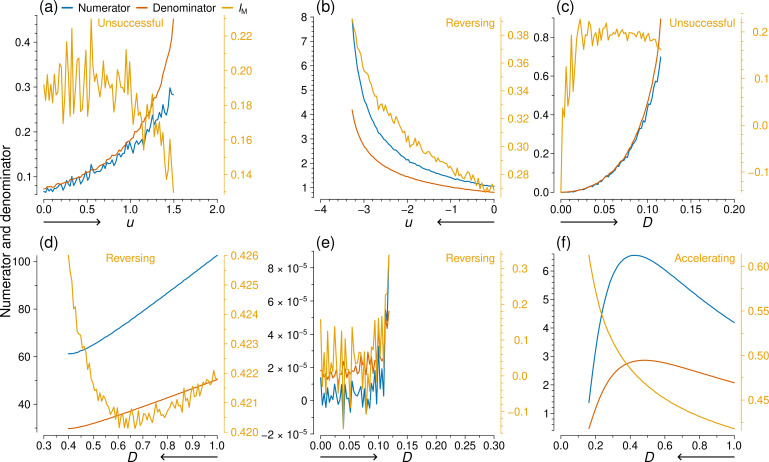
$I_{M}$ and the two quantities defining $I_{M}$ for the simulations shown in figure 5. In each panel, we show the value of $I_{M}$ (orange line), its numerator (blue line) and its denominator (red line). The direction of the simulation sequence is indicated by the arrow beneath the horizontal axis. The classification result for $I_{M}$ is shown in orange text. (a) and (b): coupled double-well, with u as the control parameter. (c) and (d): coupled double-well, with D as the control parameter. (e) and (f): SIS, with D as the control parameter.

To explore patterns in the behaviour of Moran’s I, we show in figure 6 the value of $\sum_{i=1}^{N}\sum_{j=1}^{N}A_{ij}(x_{i}-\bar{x})(x_{j}-\bar{x})/W$, which we refer to as the numerator, $\sum_{i=1}^{N}(x_{i}-\bar{x}{)}^{2}/N$, which we refer to as the denominator and $I_{M}$ for the same six series of simulations as those shown in figure 5. Note that $I_{\text{M}}$ is the ratio of the thus defined numerator to the denominator. The numerator is a covariance function. The denominator is a variance function and the same as $m_{2}$.

In figure 6a,c, both the numerator and the denominator of $I_{M}$ increase in an accelerating manner, which is desirable as an EWS. However, $I_{M}$, which is the ratio of the two quantities, at first remains around the same average value and then declines as u further increases. Therefore, the ratio of two apparently successful EWSs generates a poor EWS. In fact, $I_{M}$ is classified as unsuccessful in these two cases. By contrast, in figure 6b,e, the ratio of two increasing, accelerating quantities, which would individually make desirable EWSs, leads to a successful EWS. Conversely, in figure 6d,f, $I_{M}$ is a successful EWS, but neither of its components is. We conclude that the success or failure of $I_{M}$ depends on the intricate balance between the numerator and denominator and that $I_{M}$’s performance is not much linked to the performance of the numerator or denominator.

## 4. Discussion

We comprehensively analysed the performance of major spatial EWSs across dynamics models, control parameters, simulation directions and networks. We also gave mechanistic insights into the reasons why good performers work well under some simulation conditions but not in others (see figures 5 and 6). Figure 4, which is our main result, indicates that there is no clear overall winner. However, the figure shows some tendency, which we propose as a recommended practice. When the networked complex system shows saddle-node bifurcations (i.e. coupled double-well and mutualistic species dynamics), $g_{1}^{\prime }$ performs the best overall. Furthermore, if the system is known to transit from the lower state to the upper state, CV outperforms $g_{1}^{\prime }$. However, in ecological and deforestation dynamics, sudden large drops from an upper to lower state, which may reflect a saddle-node bifurcation, are of practical interest, corresponding to mass extinction and deforestation, respectively. Therefore, we recommend $g_{1}^{\prime }$ for these applications. By contrast, when the observables do not show sudden large jumps at tipping points (i.e. SIS and gene-regulatory dynamics), $I_{M}$ is the best at large. However, all the spatial EWSs including $I_{M}$ perform relatively poorly in two of the four simulation conditions without sudden large jumps (i.e. ascending simulations in the SIS dynamics and descending simulations with u as the control parameter in the gene-regulatory dynamics). Therefore, we conclude that tipping points of these dynamics are relatively difficult to anticipate. We emphasize that $I_{M}$, which is a commonly used spatial EWS, performs well only under specific simulation conditions (i.e. combination of dynamics with no sudden large jump, D as the control parameter, descending simulations). Furthermore, computation of $I_{M}$ needs the adjacency matrix of the network, which is not the case for CV, $g_{1}^{\prime }$ or $g_{2}$.

For the square lattice, some studies report that both spatial correlation and spatial variance anticipate tipping points acceptably well [7,22,27,39,42], whereas others report that spatial correlation outperforms spatial variance [26,37,56] or vice versa [28,43]. Still others report that alternatives such as spatial skewness and spatial kurtosis [23,29], recovery length [18,40,41], information measures [30,46,57] or a combination of measures [16] are more reliable. Other reports say that different spatial EWSs have better or worse performance even given the same dynamics model, depending on, for example, the bifurcation type [20], direction from which the bifurcation is approached [26], or parameter values [13,21,24]. We showed that spatial EWSs show a diversity of results on heterogeneous networks as well. However, we also showed that one of the two best performers on heterogeneous networks (i.e. $g_{1}^{\prime }$) performs poorly on the square lattice. Therefore, the findings available for spatial EWSs on the square lattice do not much help us understand how they behave in heterogeneous networks. This article is, to our knowledge, a first comprehensive report for heterogeneous networks. Follow-up studies using other types of spatial EWSs and a larger variety of dynamics models and heterogeneous networks, including examining the effect of network structure, are warranted for future work. Other types of spatial EWSs include spectral reddening [17], mutual information [46], spatial permutation entropy [57] and patch-size distribution and patch shape [7,19].

A side contribution of this article is a new classification method for EWSs into two successful categories and one unsuccessful category. Our proposal was motivated by the insufficiency of the predominantly used performance measure for EWSs, i.e. Kendall’s τ, which was pointed out in the literature [54,55]. Our measure aims to capture how the EWS nonlinearly changes towards a tipping point and regards that an accelerated increase nearer to the tipping point implies a better signal. However, the behaviour of EWSs is probably more diverse than what our classification or Kendall’s τ can capture. We did not provide stopping criteria either, because it is not a focus of the present work. Further work is needed for better assessments of the quality of EWSs, including temporal EWSs.

Our numerical results suggested that well-performing spatial EWSs may depend on the type of tipping points. This possibility is worth further investigation. In real applications, one does not typically know what type of tipping point is being approached. In our opinion, however, a small number of bifurcation types, in particular, saddle-node, transcritical and Hopf bifurcations, seem to be able to represent a large number of empirical systems. Therefore, we propose to focus on investigating these major types of bifurcations to further understand spatial EWSs on networks. The use of various other dynamical systems on networks and the topological normal form of dynamical systems may be fruitful to this end. Furthermore, a recently developed theory that clarified universal features of the interplay between network structure and dynamics on networks (e.g. [58–60]) may be useful for the theoretical investigation of spatial EWSs on networks. Also important is to clarify the behaviour of spatial EWSs in various false positive and false negative scenarios (e.g. when there is a regime shift or discontinuous jumps in $x_{i}$, but critical slowing down is absent; [54,57,61–63]). We have verified that the present spatial EWSs do not anticipate sudden and large jumps of $x_{i}$ in two major scenarios (described in [54,61]) in which the jumps do not involve critical slowing down. See the electronic supplementary material, S7, for the results. On the other hand, the spatial EWSs can often anticipate continuous bifurcations such as in the SIS dynamics, as expected. Because epidemic outbreaks are certainly a desired application of EWSs [64], the size or continuity of the jump should not be the only main criterion to characterize regime shifts. Furthermore, if a sudden change in the value of a system parameter induces a sudden regime shift (see the electronic supplementary material, S7), by construction, there should be no useful EWS before the parameter value changes. Therefore, we need further discussion on which type of system changes should be successfully anticipated, advancing the existing literature on false positives and negatives of EWSs [54,61].

Although spatial EWSs were originally proposed for spatial regular networks (i.e. the square lattice and its space-continuous limit), the present results suggest that these EWSs are rather more promising for heterogeneous networks, which most complex empirical networks are. Therefore, this work motivates further work on spatial EWSs for complex networks and their applications to empirical data.

## Data Availability

All data and code are available at [65]. Supplementary material is available online [66].
